# Supplementary material for: COVID-19 Management in a UK NHS Foundation Trust with a High Consequence Infectious Diseases Centre: A Retrospective Analysis
Source: Med Sci (Basel). 2021 Feb 4;9(1):6. doi: 10.3390/medsci9010006 (PMC7931073; doi:10.3390/medsci9010006)
Supplement: Supplementary file 1 [file medsci-09-00006-s001.zip › Supplementary Table S1.docx]

**Supplementary Table S1.** Cohort characteristics including univariate logistic regression analyses and associated *p* values. Odds ratios are stated per 1 unit increase in the independent variable unless otherwise stated.

|  | **N** | **Cohort (316)** | **Survived (231)** | **Died (84)** | **Univariate OR_death_ (95% CI)** | **p** |
| --- | --- | --- | --- | --- | --- | --- |
| **Age (years)** | 316 | 75 (60 – 83) | 69 (56 – 80) | 82 (76 – 89) | 1.08 (1.06 - 1.11) | <0.001 |
| **Male sex** | 316 | 173 (54.7) | 124 (53.6) | 48 (57.1) | 1.15 (0.70 - 1.91) | 0.585 |
| **White ethnicity** | 303 | 281/303 (92.7) | 203/223 (91.0) | 77/79 (97.5) | 3.79 (1.07 - 24.1) | 0.077 |
| **Symptom duration (days)** | 316 | 5 (2 – 9) | 5 (3 – 9) | 3 (1 – 7) | 0.92 (0.87 - 0.97) | 0.006 |
| **Fever** | 316 | 211 (66.8) | 165 (71.4) | 46 (54.8) | 0.48 (0.29 - 0.81) | 0.006 |
| **Cough** | 316 | 224 (70.9) | 169 (73.2) | 55 (65.5) | 0.70 (0.41 - 1.20) | 0.184 |
| **Sputum** | 316 | 76 (24.1) | 63 (27.3) | 13 (15.5) | 0.49 (0.24 - 0.92) | 0.033 |
| **Breathlessness** | 316 | 197 (62.3) | 136 (58.9) | 61 (72.6) | 1.85 (1.08 - 3.25) | 0.027 |
| **Fatigue** | 316 | 128 (40.5) | 96 (41.6) | 32 (38.1) | 0.87 (0.52 - 1.44) | 0.580 |
| **Myalgia/arthralgia** | 316 | 70 (22.2) | 59 (25.5) | 11 (13.1) | 0.44 (0.21 - 0.85) | 0.021 |
| **Diarrhoea** | 316 | 64 (20.3) | 51 (22.1) | 13 (15.5) | 0.65 (0.32 - 1.23) | 0.200 |
| **Any comorbidity^a^** | 316 | 250 (79.1) | 171 (74.0) | 78 (92.9) | 4.56 (2.03 - 12.2) | 0.001 |
| **Respiratory comorbidity^b^** | 316 | 101 (32.0) | 78 (33.8) | 23 (27.4) | 0.74 (0.42 - 1.27) | 0.284 |
| **Heart failure** | 316 | 45 (14.2) | 25 (10.8) | 20 (23.8) | 2.57 (1.33 - 4.94) | 0.004 |
| **Hypertension** | 316 | 133 (42.1) | 87 (37.7) | 45 (53.6) | 1.91 (1.15 - 3.17) | 0.012 |
| **Ischaemic heart disease** | 316 | 65 (20.6) | 44 (19.0) | 20 (23.8) | 1.33 (0.72 - 2.40) | 0.354 |
| **Chronic kidney disease** | 316 | 77 (24.4) | 49 (21.2) | 27 (32.1) | 1.76 (1.00 - 3.06) | 0.046 |
| **Diabetes mellitus** | 316 | 84 (26.6) | 58 (25.1) | 25 (29.8) | 1.26 (0.72 - 2.19) | 0.408 |
| **Active cancer** | 316 | 33 (10.4) | 23 (10.0) | 10 (11.9) | 1.22 (0.53 - 2.62) | 0.618 |
| **Immunosuppression^c^** | 316 | 27 (8.5) | 17 (7.4) | 9 (10.7) | 1.51 (0.62 - 3.46) | 0.341 |
| **Dementia** | 316 | 55 (17.4) | 29 (12.6) | 26 (31.0) | 3.12 (1.70 - 5.73) | <0.001 |
| **ACE inhibitor / ARB use** | 311 | 78/311 (25.1) | 58/227 (25.6) | 19/83 (22.9) | 0.87 (0.47 - 1.54) | 0.632 |
| **Healthcare worker** | 316 | 27 (8.5) | 27 (11.7) | 0 (0) | na | na |
| **Admitted from nursing or residential home** | 316 | 60 (19.0) | 27 (11.7) | 33 (39.3) | 4.89 (2.71 - 8.92) | <0.001 |
| **Clinical frailty score** | 311 | 4 (2 – 6) | 3 (2 – 5) | 6 (4 – 7) | 1.61 (1.40 - 1.87) | <0.001 |
| **Severe COVID-19 pneumonia** | 308 | 174/308 (56.5%) | 85/225 (37.8) | 51/83 (61.4) | 2.62 (1.57 - 4.44) | <0.001 |
| **Haemoglobin** (115-165 g/L) | 310 | 134 (115 – 145) | 135 (117 – 145) | 130 (115 – 145) | 0.95 (0.84 - 1.07)^f^ | 0.390 |
| **Platelets** (150-450 x 10^9^/L) | 298 | 217 (174 – 283) | 217 (176 – 282) | 218 (172 – 282) | 1.00 (0.97 - 1.03)^f^ | 0.972 |
| **Total WBC** (4-11 x 10^9^/L) | 311 | 7.20 (5.36 – 9.35) | 6.85 (5.21 – 8.73) | 7.97 (5.88 – 10.81) | 1.00 (0.97 - 1.02) | 0.964 |
| **Lymphocytes** (1-4 x 10^9^/L) | 311 | 0.88 (0.64 – 1.32) | 0.92 (0.65 – 1.33) | 0.82 (0.62 – 1.32) | 0.99 (0.87 - 1.07) | 0.882 |
| **Neutrophils** (2-7 x 10^9^/L) | 311 | 5.30 (3.70 – 7.48) | 5.01 (3.54 – 7.09) | 7.24 (4.37 – 9.30) | 1.17 (1.08 - 1.26) | <0.001 |
| **eGFR** (> 90 mL/min.1.73m^2^) | 307 | 68 (44 – 89) | 76 (53 - >90) | 52 (32 – 74) | 0.88 (0.83 - 0.92)^g^ | <0.001 |
| **Urea** (2.5-7.8 mmol/L) | 308 | 7.0 (4.9 – 11.4) | 6.2 (4.4 – 9.5) | 9.8 (6.5 – 16.4) | 1.10 (1.05 - 1.14) | <0.001 |
| **Sodium** (133-146 mmol/L) | 308 | 137 (134 – 140) | 137 (134 – 140) | 137 (135 – 143) | 1.07 (1.03 - 1.12) | 0.001 |
| **Potassium** (3.5-5.3 mmol/L) | 290 | 4.1 (3.8 – 4.5) | 4.1 (3.8 – 4.5) | 4.2 (3.9 – 4.6) | 1.31 (0.87 - 1.96) | 0.192 |
| **Arterial blood pH** (7.35-7.45) | 199 | 7.43 (7.39 – 7.47) | 7.44 (7.40 – 7.48) | 7.41 (7.37 – 7.45) | 0.91 (0.87 - 0.96)^h^ | 0.001 |
| **Bicarbonate** (mmol/L) | 198 | 24.8 (22.8 – 26.6) | 25.2 (23.2 – 27.5) | 23.2 (21.8 – 25.4) | 0.85 (0.77 - 0.93) | <0.001 |
| **CRP** (<5 mg/L) | 306 | 72 (30 – 131) | 65 (23 – 119) | 90 (50 – 176) | 1.04 (1.01 - 1.06)^f^ | 0.007 |
| **ALT** (0-40 U/L) | 263 | 24 (15 – 39) | 26 (16 – 40) | 18 (13 – 28) | 1.01 (0.99 - 1.03)^g^ | 0.344 |
| **ALP** (30-130 IU/L) | 285 | 78 (63 – 102) | 76 (62 – 98) | 84 (66 – 110) | 1.02 (1.00 - 1.04)^g^ | 0.025 |
| **Bilirubin** (0-21 µmol/L) | 296 | 9 (6 – 12) | 9 (7 – 11) | 9 (6 – 14) | 1.03 (1.00 - 1.08) | 0.082 |
| **Albumin** (35-50 g/L) | 298 | 38 (35 – 41) | 39 (36 – 42) | 37 (34 – 40) | 0.92 (0.87 - 0.97) | 0.003 |
| **Heart rate** | 307 | 89 (77 – 105) | 89 (76 – 105) | 90 (79 – 107) | 1.02 (0.96 - 1.08)^g^ | 0.548 |
| **Systolic blood pressure** | 307 | 126 (111 – 140) | 125 (110 – 139) | 130 (113 – 140) | 1.04 (0.98 - 1.09)^g^ | 0.191 |
| **Diastolic blood pressure** | 307 | 72 (64 – 81) | 73 (65 – 82) | 69 (63 – 76) | 0.94 (0.86 - 1.03)^g^ | 0.209 |
| **Respiratory rate** | 306 | 21 (18 – 26) | 20 (18 – 24) | 24 (18 – 30) | 1.09 (1.04 - 1.14) | <0.001 |
| **Oxygen saturations prior to oxygen therapy** | 226 | 95 (91 – 97) | 95 (92 – 97) | 93 (88 – 96) | 0.95 (0.91 - 0.98) | 0.003 |
| **Hypoxia^d^** | 308 | 192/308 (62.3) | 129/225 (57.3) | 63/83 (75.9) | 2.75 (1.64 - 4.64) | <0.001 |
| **Temperature** | 309 | 37.2 (36.6 – 37.9) | 37.2 (36.6 – 37.9) | 37.1 (36.3 – 37.7) | 0.79 (0.59 - 1.04) | 0.103 |
| **Definite COVID-19 on baseline CXR** | 303 | 121/303 (39.9) | 88/219 (40.2) | 32/83 (38.6) | 0.93 (0.55 - 1.56) | 0.796 |
| **CURB65 score^e^** | 299 | 2 (1 – 2) | 1 (0 – 2) | 2 (1 – 3) | 2.20 (1.67 - 2.95) | <0.001 |

Data are median (IQR) for continuous variables, and n (%) (or n/N (%)) for categorical variables. Local laboratory normal ranges for blood tests are shown in parentheses. ^a^Presence of at least one of respiratory comorbidity, heart failure, diabetes, active cancer or immunosuppression. ^b^Defined as at least one of: asthma, chronic obstructive pulmonary disease (COPD), interstitial lung disease, obstructive sleep apnoea, home nebuliser/oxygen/non-invasive pressure support. ^c^Defined as at least one of: immunodeficiency syndrome, maintenance steroids (prednisolone ≥ 5mg/day, hydrocortisone ≥ 15mg/day, any dose dexamethasone); conventional synthetic immunosuppressive drugs (excluding hydroxychloroquine and sulfasalazine); biologics; Janus kinase (JAK) inhibitors; cytotoxic chemotherapy within past 6 months. ^d^Defined as oxygen saturations ≤ 94% on room air, or any use of supplemental oxygen. ^e^CURB65 with 1 point each for confusion, urea > 7, respiratory rate > 30, systolic blood pressure < 90 mmHg or 60 mmHg diastolic, and age ≥ 65. ^f^OR stated per 10 unit increase in the independent variable. ^g^OR stated per 5 unit increase in the independent variable. ^h^OR stated per 0.01 unit increase in arterial blood pH.

ACE: angiotensin converting enzyme, ALP: alkaline phosphatase, ALT alanine aminotransferase, ARB: angiotensin receptor blocker, CRP: C-reactive protein, COPD: chronic obstructive pulmonary disease, CXR: chest x-ray, eGFR: estimated glomerular filtration rate, IQR: interquartile range, N: total number of measurements for each variable for the entire cohort, OR: odds ratio, WBC: white blood cell.
